# Automated Quantitative Lung CT Improves Prognostication in Non-ICU COVID-19 Patients beyond Conventional Biomarkers of Disease

**DOI:** 10.3390/diagnostics11112125

**Published:** 2021-11-16

**Authors:** Pierpaolo Palumbo, Maria Michela Palumbo, Federico Bruno, Giovanna Picchi, Antonio Iacopino, Chiara Acanfora, Ferruccio Sgalambro, Francesco Arrigoni, Arturo Ciccullo, Benedetta Cosimini, Alessandra Splendiani, Antonio Barile, Francesco Masedu, Alessandro Grimaldi, Ernesto Di Cesare, Carlo Masciocchi

**Affiliations:** 1 Department of Diagnostic Imaging, Area of Cardiovascular and Interventional Imaging, Abruzzo Health Unit 1, Via Saragat, Località Campo di Pile, 67100 L’Aquila, Italy; arrigoni.francesco@gmail.com; 2 Italian Society of Medical and Interventional Radiology (SIRM), SIRM Foundation, 20122 Milan, Italy; federico.bruno.1988@gmail.com; 3 Department of Anesthesiology and Intensive Care Medicine, Fondazione Policlinico Universitario A. Gemelli IRCCS, Catholic University of The Sacred Heart, 00168 Rome, Italy; michelapalumbo85@gmail.com; 4 Department of Applied Clinical Sciences and Biotechnology, University of L’Aquila, Via Vetoio 1, 67100 L’Aquila, Italy; ant.iacopino@gmail.com (A.I.); acanforachiara@gmail.com (C.A.); ferrucciosgalambro@gmail.com (F.S.); alessandra.splendiani@univaq.it (A.S.); francesco.masedu@univaq.it (F.M.); carlo.masciocchi@univaq.it (C.M.); 5 Infectious Disease Unit, San Salvatore Hospital, Via Lorenzo Natali, 1-Località Coppito, 67100 L’Aquila, Italy; giovanna.picchi.inf@gmail.com (G.P.); arturo.ciccullo@gmail.com (A.C.); a.grimaldi@gmail.com (A.G.); 6 Department of Life, Health and Environmental Sciences, University of L’Aquila, Piazzale Salvatore Tommasi 1, 67100 L’Aquila, Italy; benedetta.cosimini@hotmail.it (B.C.); ernesto.dicesare@univaq.it (E.D.C.)

**Keywords:** COVID-19, lung inflammation, prognosis, tomography computed scanners, lung volume measurement

## Abstract

(1) Background: COVID-19 continues to represent a worrying pandemic. Despite the high percentage of non-severe illness, a wide clinical variability is often reported in real-world practice. Accurate predictors of disease aggressiveness, however, are still lacking. The purpose of our study was to evaluate the impact of quantitative analysis of lung computed tomography (CT) on non-intensive care unit (ICU) COVID-19 patients’ prognostication; (2) Methods: Our historical prospective study included fifty-five COVID-19 patients consecutively submitted to unenhanced lung CT. Primary outcomes were recorded during hospitalization, including composite ICU admission for the need of mechanical ventilation and/or death occurrence. CT examinations were retrospectively evaluated to automatically calculate differently aerated lung tissues (i.e., overinflated, well-aerated, poorly aerated, and non-aerated tissue). Scores based on the percentage of lung weight and volume were also calculated; (3) Results: Patients who reported disease progression showed lower total lung volume. Inflammatory indices correlated with indices of respiratory failure and high-density areas. Moreover, non-aerated and poorly aerated lung tissue resulted significantly higher in patients with disease progression. Notably, non-aerated lung tissue was independently associated with disease progression (HR: 1.02; *p*-value: 0.046). When different predictive models including clinical, laboratoristic, and CT findings were analyzed, the best predictive validity was reached by the model that included non-aerated tissue (C-index: 0.97; *p*-value: 0.0001); (4) Conclusions: Quantitative lung CT offers wide advantages in COVID-19 disease stratification. Non-aerated lung tissue is more likely to occur with severe inflammation status, turning out to be a strong predictor for disease aggressiveness; therefore, it should be included in the predictive model of COVID-19 patients.

## 1. Introduction

More than a year after the first cases in Wuhan, COVID-19 continues to represent a worrying pandemic considering numbers and real-world variability. Despite a broader representation of asymptomatic or paucisymptomatic cases, COVID-19 can lead to severe illness in up to 14% of patients. Moreover, 5% become critical with 49% of mortality in some case series [1]. Thus, identifying patients with potential for severe or critical illness should be considered of primary importance.

Several studies have tried to identify potential diagnostic and predictive models in the COVID-19 approach, unfortunately often reporting a high risk of bias [2,3,4]. Therefore, the clinical validity of different predictors remains undetermined. 

There are different reasons for clinical uncertainties and therapeutic failure in COVID-19. In fact, recent evidence has reconsidered the pathobiogenesis of COVID-19, shifting the attention from a dominant primary lung injury to all vasculitis-like anomalies and immune-mediated thrombosis [5,6,7,8,9,10,11,12]. 

Diagnostic imaging also has an uncertain role [13,14,15,16,17,18,19]. Evidence of this can be found in the current World Health Organization (WHO) recommendations on chest imaging in the diagnosis and management of COVID-19, which are based only on low-level evidence or expert recommendations, meaning without significant support from the literature [20]. 

In this scenario, WHO recommendations suggest chest imaging mainly as support to clinical and laboratory assessment to decide the most adequate management of COVID-19 patients, although nonquantitative parameters to be considered are provided [20]. 

Among different imaging tools, computed tomography (CT) can offer significant advantages in risk stratification. 

Lung CT showed indeed the highest sensitivity in identifying interstitial involvement [21,22,23,24,25,26,27]. In a recent meta-analysis by Xu et al., including 3235 patients, CT reached a pooled sensitivity of 92%, although a low specificity [28]. CT imaging cannot identify COVID-19 without lung involvement, although specific imaging biomarkers are proposed to improve disease differentiation [29,30]. 

Lung CT finds encouraging results also in describing the disease, quantifying the burden of disease and as a disease-progression predictor, beyond conventional clinical and respiratory parameters used in real-world practice. 

Different analysis methods have been applied in clinical practice, most of them based on qualitative (i.e., description of lesion characteristics including ground glass opacities or consolidation) or semiquantitative analysis (i.e., severity score or overall score based on percentage of different lobe or segment involvement). 

Multiple papers highlight a significant correlation between severity score and a more aggressive disease [31,32]. Moreover, Artificial Intelligence (AI) improves the detection of “heat” areas able to stage disease progression [33,34,35,36,37].

More recently, lesion volume measurement through the identification of different densitometric thresholds, as a quantitative method, has become a promising approach to COVID-19 [38,39]. 

**Figure 1:**
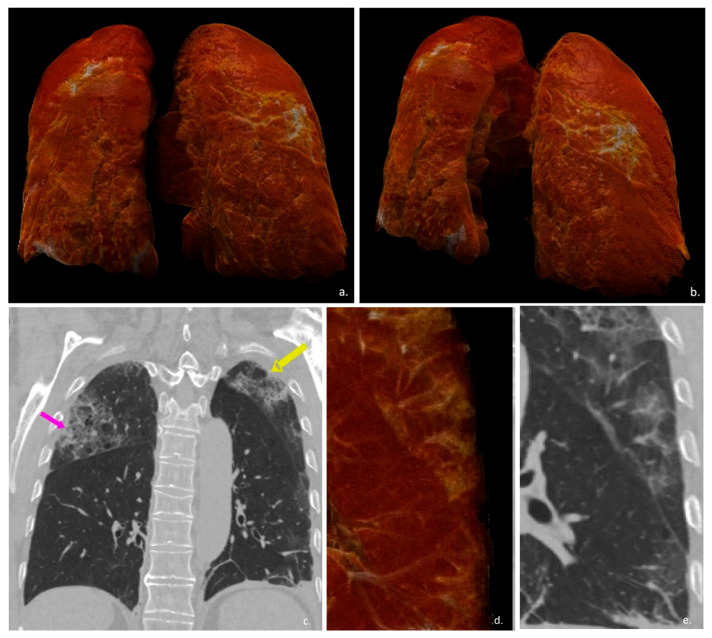
Automatic lung volume detection. Two different views from a volume-rendered reconstruction were obtained from the automatic detection of lung tissue (img (**a**,**b**)). Pink and yellow arrows in img (**c**) highlight also high-density lesion within lung tissue, in a coronal view. In images (**d**,**e**), magnified, volume-rendered reconstruction and coronal CT scan view (respectively) enhance the high capability of CT and quantitative reconstruction to detect also millimetric lesion.

Unlike the conventional qualitative or semiquantitative lung analysis, quantitative lung CT allows identification of lung volumes and weight, differentiating non- or poorly aerated areas that can differently influence gas exchange (Figure 1) [31,32,40,41,42,43,44]. 

In a recent paper of Chiumello et al., quantitative CT patterns resulted advantageous also in describing COVID-19 as a nonconventional subset of acute respiratory distress syndrome (ARDS) when compared to lung physiology [39].

Different studies also showed the effectiveness of quantitative lung assessment in prediction of disease progression, limiting the analysis to the volumetric quantification of lesion. 

However, lung tissue weight can be derived from quantitative analysis through volume and mean density assessment, thus allowing a specific differentiation between areas with different gas/tissue ratios, which can turn critically in the clinical work-up of a symptomatic COVID-19 patient [39,45,46,47,48,49,50]. 

Going back to these premises, the purpose of our study was to evaluate the impact of quantitative lung CT including volumetric and tissue weight assessment of differently aerated areas of lung on non-intensive care unit (ICU) COVID-19 patients’ prognostication.

## 2. Materials and Methods

This study was carried out after the approval of our university’s Internal Review Board committee. This is a retrospective assessment of prospectively followed-up patients (historical prospective study). 

**Figure 2:**
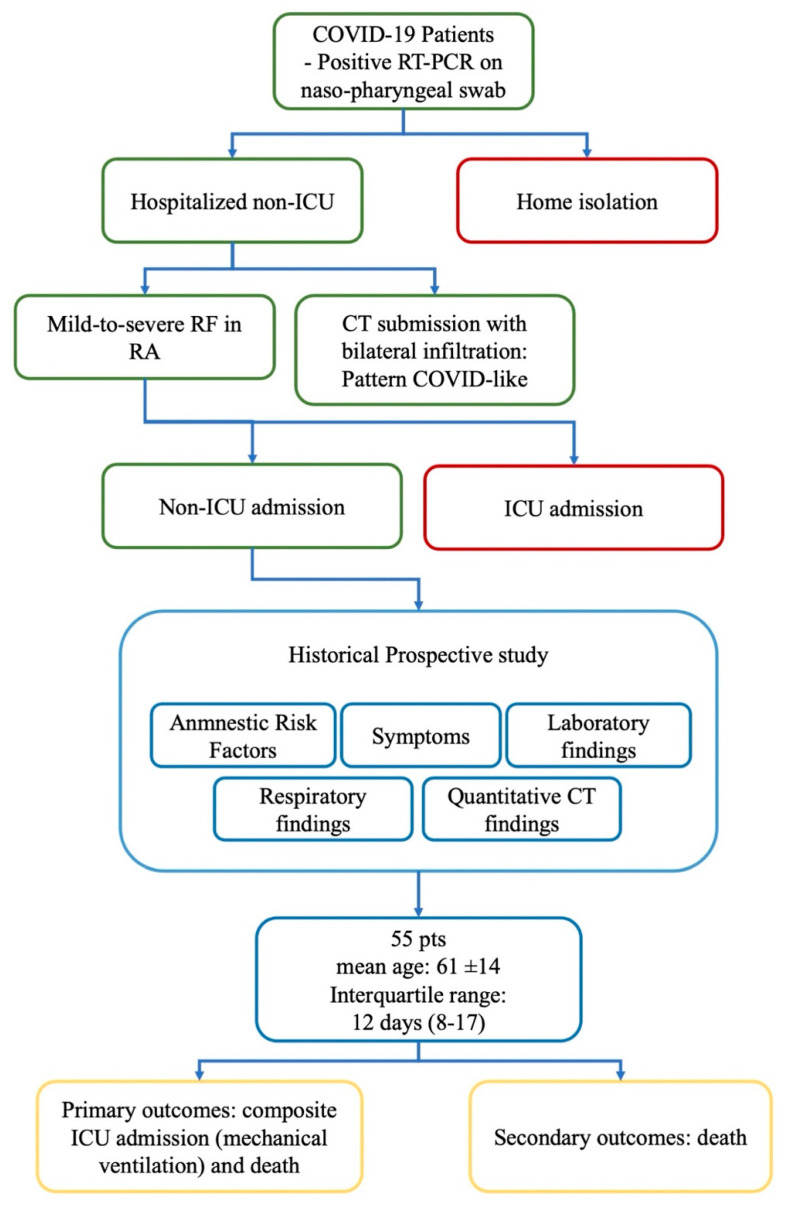
Flowchart of the study. Green rectangles show inclusion criteria; in red, exclusion criteria. Analyzed parameters are within blue rectangles. In yellow, outcomes. RT-PCR: reverse transcriptase polymerase chain reaction; ICU: intensive care unit; RF: respiratory failure; RA: room air; CT: computed tomography; pts: patients.

All patients had documented COVID-19 (i.e., positive reverse transcriptase polymerase chain reaction (RT-PCR) on nasal or pharyngeal swab). Hospitalized non-ICU COVID-19 patients submitted to lung CT for an adequate work-up at admission were included. Figure 2 shows the flowchart of our study.

### 2.1. Exam Protocol

Unenhanced CT examinations were performed with a Canon Aquilion One (320 rows detectors, 0.5 mm collimation; Canon Medical Systems, Otawara, Japan) (120 kv, ADE; mean dose < 5 mSv). All examinations were acquired at room air (RA). Whole-lung CT was performed under static conditions during an end-inspiratory hold. CT volumes were reconstructed in both lung and mediastinal windows (W: 1600 L: −550 and W: 40A0 L: 40, respectively).

### 2.2. Postprocessing Analysis

Postprocessing analysis was performed with dedicated software (CT Lung Density Analysis, Vitrea Advance Visualization, Canon) [51]. Notably, an automated segmentation of lung tissues with quantifiable controls and renderings is performed by the software. The Lung Density Analysis Tool segments the airways (including the trachea, main bronchi, and some larger bronchioles) and vascular structures, for both left and right lung. The contours of all the segmented structure are highlighted with different colors, and the possibility to edit contours for corrections is enabled. 

Lung tissue and Hilary structures were manually corrected after automatic segmentation registration if errors or inaccuracy were detected. 

**Figure 3:**
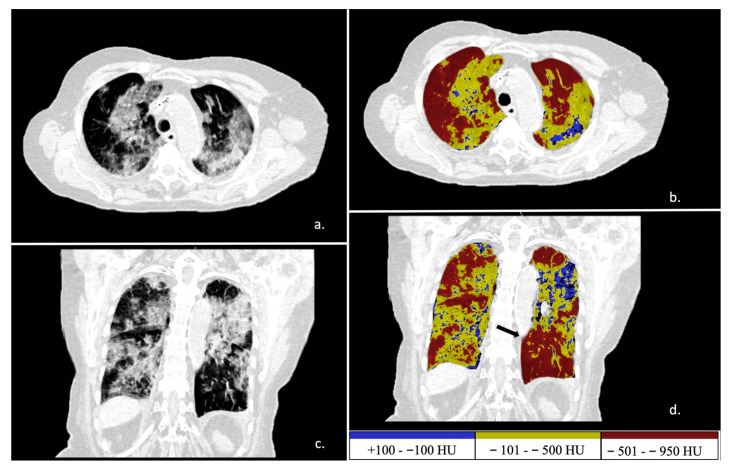
Automatic tissue density analysis. In images (**a**,**b**), axial views. In images (**c**,**d**), coronal views. In images (**a**,**c**), unenhanced CT slices of an extensive lung COVID-19 involvement with bilateral infiltration. In images (**b**,**d**), the software-dedicated analysis differentiated non-aerated (blue) and poorly aerated (yellow) tissue. In red, well-aerated lung tissue. In image (**d**), the black arrow indicates the exclusion of Hilary structure from high-density tissue. Color bar values on the bottom right.

Different volumes were analyzed by setting specific densitometric ranges within total lung tissue, i.e., overinflated tissue (density < −950 Hounsfield Unit—HU), well-aerated tissue (ranging from −950 to −501 HU), poorly aerated tissue (ranging from −500 to −101 HU), and non-aerated tissue (ranging from −100 to +100 HU) (Figure 3).

The total lung tissue volume is computed using all segmented lung voxels, including unclassified voxels, that is, lung voxels outside the defined HU ranges.

The analysis was performed on total volume to avoid partial volume artifact. 

Calculation of weight of differently aerated areas was made through volumes and mean density (lung weight = lung volume × (mean density + 1000)/1000) [45,46,47]. 

Moreover, in a subset of ten examinations, lung volumes were compared with data obtained via an AI-based software [30]. 

### 2.3. Clinical Follow-Up Study

All patients were clinically followed up during their hospitalization in the Infectious Disease Clinic of our hospital. Symptoms, clinical risk factors, laboratory, and respiratory data were collected at admission and during hospitalization. Primary outcomes were composite ICU admission for the need of mechanical ventilation and/or death occurrence. Secondary outcomes considered death occurrence.

### 2.4. Statistical Analysis

Descriptive variables are presented as mean and correspondent confidential intervals or as percentages (frequencies). The Shapiro–Wilk (SW) test was used to evaluate data distribution. The distributional assumption for parametric analysis was fulfilled according to the SW test. A *t*-test was used for normal variables comparison; a chi-squared test was used with nominal (dichotomic) variables. Cox regression analysis was used to test the predictive validity of quantitative CT parameters. Variance inflation factor (VIF) was considered for evaluating multicollinearity. Kaplan–Maier was tested for qualitatively evaluating outcomes fitting for patients categorized for the median of the best predictor. 

Differences between inter-software volume detection were tested with an independent-sample *t*-test. Bland–Altman analysis with 95% limits of agreement was used for inter-software agreement in assessment of total lung volumes and high-density volumes; differences are plotted as percentage.

To investigate the added value of quantitative parameters to predict disease progression, the following 4 models were used: Model I (only clinical characteristics), Model II (Model I + respiratory and laboratoristic characteristics), Model III (Model II + percentage of non-aerated lung volume), and Model IV (Model II + percentage of non-aerated tissue-weight). As a measure of discrimination, we calculated the area under the receiver-operating characteristics curve (C-index) with 95% confidence intervals (CIs) for diagnosis of significant disease progression. The added value of Models II, III, and IV beyond the basic model was quantified by the change in the C-index.

An alpha error of 5% was used as a threshold of significance, conventionally considered as an acceptable threshold for a conditional probability of 5% to experience a type I error. All statistical analyses were performed with SPSS (IBM Corp. Released 2016. IBM SPSS Statistics for Mac, Version 26.0. Armonk, NY, USA: IBM Corp). 

## 3. Results

### 3.1. Patient Population

Fifty-five patients (mean age 61 ± 14 years; 37 males) met inclusion criteria, i.e., patients consecutively admitted to the Infectious Disease Clinic of our hospital with (i) an RT-PCR-based diagnosis of COVID-19; (ii) respiratory failure in RA that did not require mechanical ventilation; (iii) CT with typical COVID-like pattern and bilateral infiltrations. 

Baseline characteristics of the patient population are described in Table 1. 

In a mean follow-up of 12 days (interquartile range: from 8 to 17), eleven patients reported primary outcomes, and in four out of eleven patients, death occurred.

Patients with disease progression showed a higher percentage of obesity, hypertension, diabetes, and chronic obstructive pulmonary disease (COPD). Moreover, they were more often dyspneic. Fever was the most reported clinical sign (52 out of 55 patients, 95%, reported fever). 

Antiviral drugs were administered to almost all patients (52 out of 55, 95%: *p*-value 0.006 in patients categorized for primary outcomes).

Conversely, steroid medications were reported in 44% only (*p*-value 0.124). 

Lastly, heparin was reported in 78% of patients (*p*-value 0.551). 

### 3.2. Laboratory and Respiratory Findings

Among different laboratory findings, neutrophile-to-leucocyte ratio (NLR), lactate dehydrogenase (LDH), and D-Dimer were significantly higher in patients with disease progression. Among respiratory data, SpO_2_ and PF resulted significantly different between the two groups (Table 1). 

### 3.3. Quantitative Lung CT: Inter-Software Agreement 

A nonsignificant difference was observed between total lung volume calculated with different software (4642 ± 1069 vs. 4632 ± 1297 mL; *p*-value 0.985). Similar evidence was highlighted for differently aerated tissue subanalysis (50 ± 36 vs. 60 ± 45 mL of non-aerated lung volume: *p*-value 0.584; 290 ± 142 vs. 305 ± 132 mL of poorly aerated lung volume: *p*-value 0.806; 4033 ± 1052 vs. 3879 ± 1223 mL of well-aerated lung volume: *p*-value 0.767; 269 ± 186 vs. 326 ± 278 mL of overinflated lung volume: *p*-value 0.594). 

**Figure 4:**
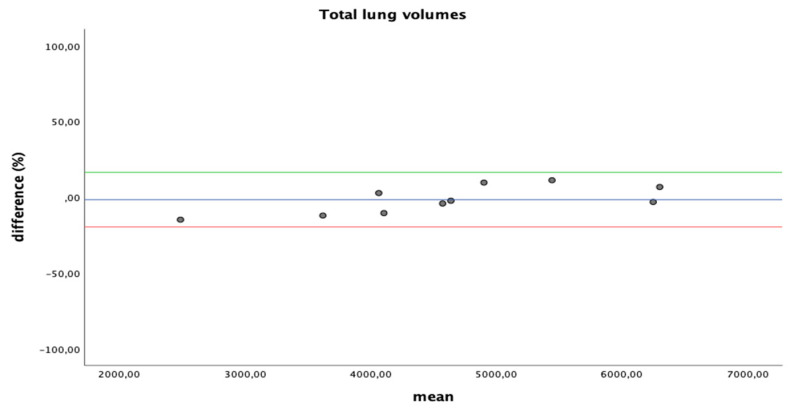

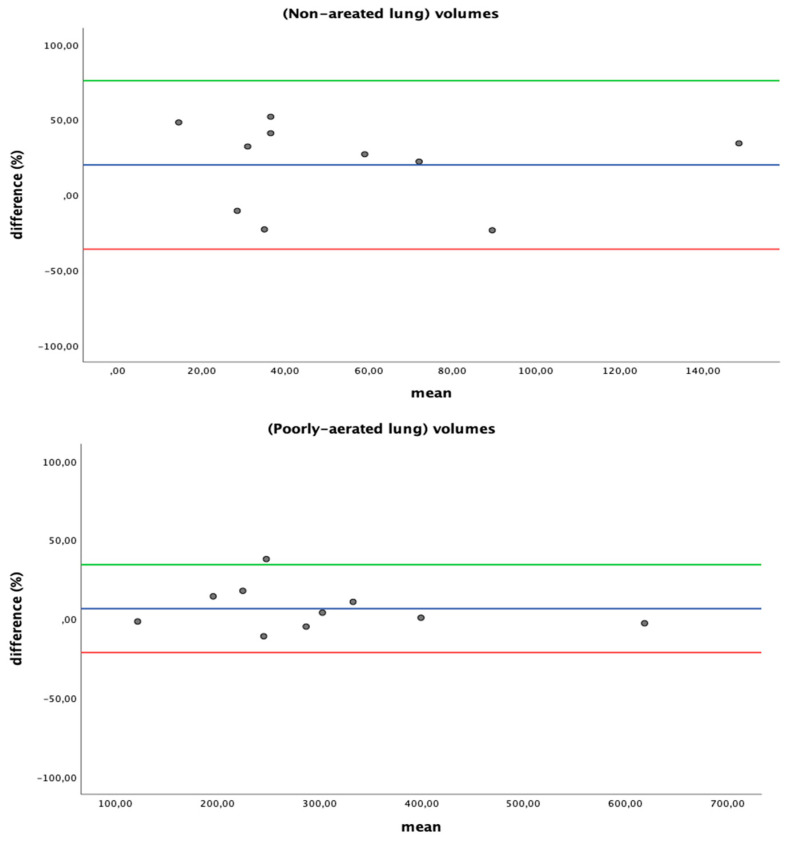
Bland–Altman plots for agreement between different readings; differences are plotted as percentage.

Bland–Altman plots of the agreement between different measure for total and high-density volumes are shown in Figure 4.

### 3.4. Quantitative Lung CT: Lung Parameter

Patients who reported disease progression showed lower total lung volume despite similar weights. Moreover, non-aerated and poorly aerated lung tissue resulted significantly higher in patients with disease progression (*p*-value 0.003 and 0.011, respectively); well-aerated tissue resulted lower (*p*-value 0.011). 

Conversely, overinflated tissue was similar between the two groups. 

Scores of non-aerated tissue expressed as a percentage of total weight (NAw%) and percentage of total volume (NAv%) were also calculated, both of which resulted higher in patients with disease progression (0.001 and 0.003, respectively). Similarly, high-density tissue, including both non-aerated and poorly aerated tissue, resulted higher in patients with disease progression when calculated as weight and volume percentage (HDw% and HDv%, *p*-value 0.001 and 0.002, respectively) (Table 1). 

### 3.5. Comparison between CT Parameters and Laboratory/Respiratory Findings

Good correlation resulted when laboratory findings were compared with CT parameters, except for overinflated tissue. 

Among respiratory findings, PaO_2_ and SpO_2_ did not correlate with CT parameters; conversely, PF correlated with CT parameters, except for overinflated tissue (Table 2). 

D-Dimer and PF showed an inverse correlation (r −0.46, *p*-value 0.001).

Moreover, NLR, D-Dimer, and LDH correlate mainly with high-density tissues. All three parameters resulted as predictors of non-aerated tissue in linear regression analysis, although NLR only was independently associated with higher non-aerated lung tissue in a multivariate regression analysis (*p*-value 0.0001).

### 3.6. Predictive Validity of Quantitative CT Parameters and Association with Outcomes

All CT parameters except total lung weight and overinflated tissue showed good predictive validity in a univariate analysis. When CT parameters were compared (avoiding linearly dependent covariates: well-aerated tissue and lung volumes showed high VIF, i.e., more than 10), non-aerated lung tissue only showed independent predictive validity. 

NAw% resulted an independent predictor of disease progression compared to NAv% (both with low VIF) and other variables (i.e., D-dimer, NLR, and PF, which resulted predictive of poor prognosis in the univariate analysis) (Table 3).

**Figure 5:**
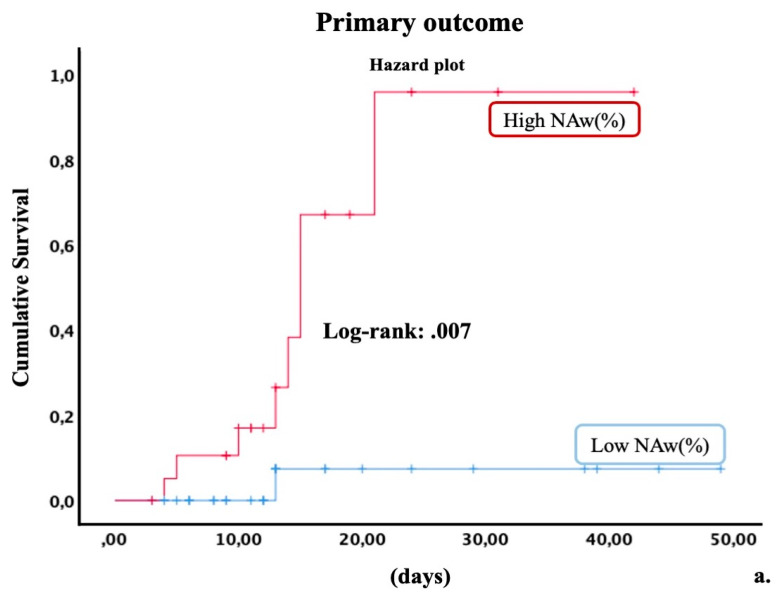

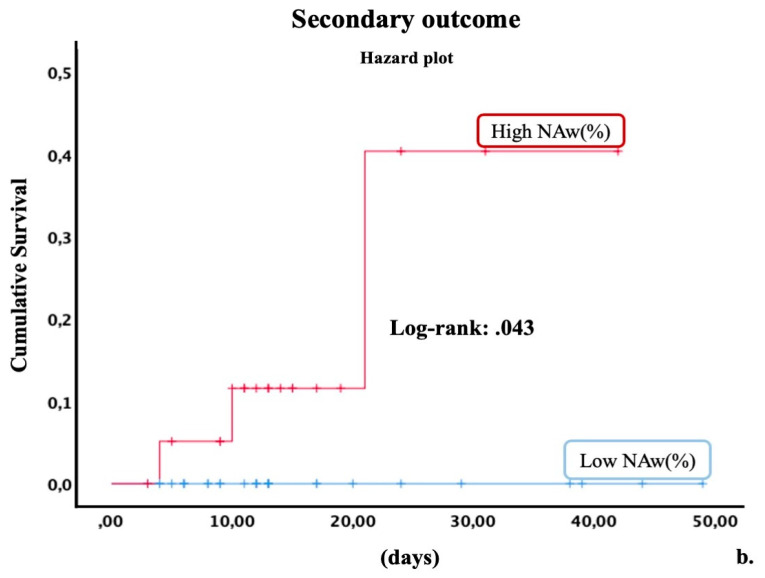
Kaplan–Meier analysis. Cumulative risk for primary (**a**) and secondary (**b**) outcome in patients categorized for median NAw (%) (percentage of non-aerated tissue weight).

When patients were categorized for median NAw%, Kaplan–Meier’s analysis showed a significant risk of primary and secondary outcomes in patients with higher NAw% (Figure 5).

**Figure 6:**
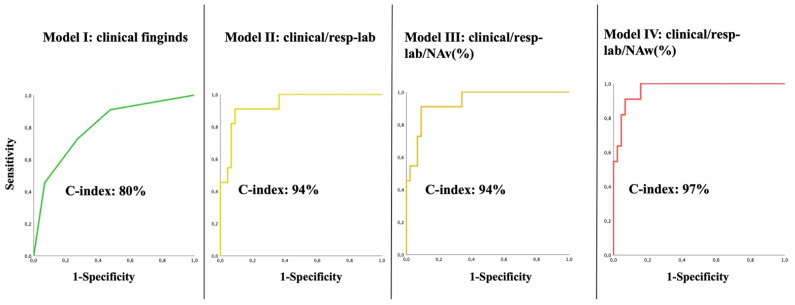
ROC analysis for Models I, II, III, and IV. Model I (clinical findings; green line) exhibited the lowest C-index (0.8; 95% CI: 0.66–95). Models II and III (Model I + respiratory/laboratoristic findings and Model II + NAv (%), respectively; yellow and orange lines) showed a similar C-index (0.94; 95% CI: 0.86–1). Model IV (Model II + NAw (%); red line) showed the highest C-index (0.97; 95% CI: 0.93–1). NAv (%): percentage of non-aerated lung volume; NAw (%): percentage of non-aerated tissue-weight.

A predictive model including clinical factors only (Model I) resulted in a C-index of 0.80 (95% CI: 0.66–95; standard error: 0.08; *p*-value 0.002). When laboratory and respiratory findings were added (Model II), C-index increased to 0.94 (95% CI: 0.86–1; standard error: 0.04; *p*-value 0.0001). When NAv (%) was added (Model III), no difference was observed. Conversely, when NAw (%) was added (Model IV), C-index increased to 0.97 (95% CI: 0.93–1; standard error: 0.02; *p*-value 0.0001) (Figure 6). 

## 4. Discussion

Our historical prospective study included hospitalized non-ICU COVID-19 patients.

Our analysis highlights some critical findings:

(i) Quantitative lung CT allows accurate staging of the severity of COVID-19 pneumonia beyond the extension of infiltration on which current severity score is based; 

(ii) Risk modeling of non-ICU COVID-19 patients reached the best C-index once it included non-aerated tissue over conventional risk stratification based on clinical, laboratoristic, and respiratory findings.

Different studies have still shown critical advantages of quantitative assessment of COVID-19 [52,53]. 

First, quantification of lung volumes with lower total and well-aerated lung tissue in patients with disease progression than in patients discharged (as in our case series) turned out to be a key factor in setting some mechanical ventilation parameters [39,49,54,55,56,57,58,59].

Moreover, quantitative lung CT at admission also resulted effective in the short-term risk stratification of COVID-19 patients for disease progression, i.e., mechanical ventilation and/or death [60,61].

Colombi et al. highlighted the predictive validity of visual semiquantitative or automatic quantitative analysis of the extension of the well-aerated lung on a patient population of 236 [62]. Similarly, Lanza et al. showed the advantages of high-density lung quantification (including non-aerated and poorly aerated areas) in stratifying COVID-19 patients on 222 participants [63]. 

However, volumetric quantification suffers from the inability to specifically differentiate areas with different gas/tissue ratios, i.e., to adequately differentiate non-aerated (0% gas) and poorly aerated (50% gas and 50% tissue) lung tissue.

This discrimination is logically critical.

Progressive respiratory failure develops in many COVID-19 patients, with severe illness reported in 14% of cases, in some case series [1]. 

Although different clinical risk factors were associated with severe and critical COVID-19, clinical evolution often remains unpredictable, and an accurate biomarker of disease aggressiveness is lacking [64]. 

Clinical variability depends on different interactive pathological mechanisms. 

In fact, COVID-19 showed different patterns, which distinguished the pulmonary pathobiology of COVID-19 from that of equally virus infection [65,66]. 

Recent theories on COVID-19 pathophysiology have focused on vasculitis-like anomalies or immune-mediated thrombosis in which viral alveolitis drives inflammation, endotheliopathy, and microvascular injury, leading to vascular tone dysregulation and pulmonary intravascular coagulopathy [5,6,7,8,9,10,11,12]. Postmortem reports and pathological specimens from COVID-19 patients showed DAD together with pulmonary infarction, small pulmonary vessel and capillary thrombosis, and hemorrhage [65,67,68,69,70,71,72]. 

In confirmation of this, in a previous work of Chiumello et al., a weak correlation between the venous admixture (cause of hypoxemia) and non-aerated tissue suggested that the major component of the venous admixture in COVID-19 pneumonia is the ventilation–perfusion mismatch rather than the true right-to-left shunt due to nonventilated consolidated tissues (unlike what was previously observed in typical ARDS) [39,42,43,49,73,74].

This evidence is consistent with the presence of ground-glass opacities (with or without localized pulmonary consolidations) and the immune-driven intrapulmonary thrombosis since the early phase of the disease in a lung with preserved mechanics [39,48,49,50,73].

In our case series also, PF and high-density tissue showed only a weak correlation. In contrast, an almost moderate correlation was found between D-Dimer and PF and between D-Dimer and high-density tissues, supporting the hypothesis of the presence of a hypercoagulable state.

Moreover, D-dimer and NLR—as also reported in literature—correlate with the severity of inflammation, a poor prognosis, and fatal outcome [71,75,76,77,78,79].

The predictive relationship observed between D-Dimer, LDH, and NLR with non-aerated tissue in our results suggests that the latter is more likely to occur with a severe inflammation status in a later phase of the disease when consolidation and fibrotic-like changes prevail [80]. 

Probably due to these considerations, non-aerated tissue showed an independent predictive validity on patient outcome and was the best index of disease progression, compared to the other tissues and covariates.

This evidence was also confirmed when tissue volume and weight were compared, showing the independent predictive validity of the latter probably related to a greater capability in discriminating disease activity over the extension of disease. Supporting this, the C-index of the model including non-aerated tissue reached 97%, while it did not vary substantially when extension (volume) only was considered. 

Therefore, modeling the patient’s risk by quantifying non-aerated tissue over clinical and respiratory findings results highly effective. 

This marker resulted effective despite the following: 

(i) The inability of CT to differentiate consolidation and potentially recruitable atelectasis within the non-aerated lung tissue [81]; 

(ii) The early CT pattern showed in our case series, considering the observed percentage of pulmonary involvement and NAw (%).

(iii) The dichotomy between the dynamic nature of COVID-19 disease and the capability of CT to provide only anatomical information at the time of acquisition. However, the predictive model resulted effective especially when both clinical information and quantitative CT parameters were considered, i.e., in patients with moderate-to-severe symptoms who required hospitalization, thus confirming its prevalent role in the patients’ management beyond conventional risk prediction. 

This study had several limitations: (i) it was a single-center retrospective analysis; (ii) there was a limited sample for the inter-rater agreement for software-based quantification; however, this is an automatic quantification based on densitometric values and is therefore expected to remain high also with an increased sample; (iii) there was a limited sample size.

## 5. Conclusions

COVID-19 represents a critical issue in the global health system [82,83,84,85,86].

An accurate modeling of prediction of disease aggressiveness continues to be a cornerstone in the approach to COVID-19. 

Quantitative lung CT provides wide advantages in disease stratification of patients with moderate-to-severe symptoms requiring hospitalization, offering significant insight into COVID-19 lung disease through the automatic detection of high-density areas.

Notably, non-aerated lung tissue mainly showed a strong predictive validity for disease progression to mechanical ventilation or death and therefore should be included in the prognostic model of COVID-19 patients.

## Figures and Tables

**Table 1:** Baseline characteristic of patient population.

		All (n 55)	Discharge	Worst Outcome	*p*-Value
Age		61 ± 14	62 ± 11	61 ± 16	0.821
Male n (%)		37 (67)	30 (55)	7 (12)	0.518
Female n (%)		21 (38)	14 (25)	4 (7)	
***Clinical characteristics***					
Obesity n (%)		19 (35)	12 (22)	7 (13)	0.03 *
Hypertension n (%)		31 (56)	22 (40)	9 (16)	0.056
Diabetes n (%)		10 (18)	6 (11)	4 (7)	0.099
CVD n (%)		14 (25)	10 (18)	4 (7)	0.285
CKF n (%)		3 (5)	1 (2)	2 (3)	0.099
Cerebrovascular disease n (%)		4 (7)	3 (5)	1 (2)	0.602
COPD n (%)		6 (11)	3 (5)	3 (5)	0.087
Asthma n (%)		1 (2)	1 (2)	0	0.8
Epathopathy n (%)		5 (9)	3 (5)	2 (4)	0.259
Neoplasia n (%)		5 (9)	3 (5)	2 (4)	0.259
Smocking habits (%)		15 (27)	10 (18)	5 (9)	0.109
Fever n (%)		52 (95)	43 (78)	9 (16)	0.099
Rhinitis n (%)		2 (4)	2 (4)	0	0.637
Conjunctivitis n (%)		6 (11)	4 (7)	2 (4)	0.344
Anosmia n (%)		7 (13)	5 (9)	2 (4)	0.429
Pharyngodynia n (%)		6 (11)	4 (7)	2 (4)	0.344
Cough n (%)		32 (58)	25 (45)	7 (13)	0.478
Dyspnea n (%)		20 (36)	12 (22)	8 (15)	0.008 **
Arthromyalgia n (%)		6 (11)	6 (11)	0	0.244
Asthenia n (%)		8 (15)	6 (11)	2 (4)	0.508
Syncope n (%)		2 (4)	2 (4)	0	0.637
GI n (%)		11 (20)	9 (16)	2 (4)	0.618
***Laboratory and respiratory characteristics at admission***					
Neutrophils 103/µL		4 ± 1.8	4 ± 1.9	5 ± 2.9	0.304
Lymphocytes (SI)		1 ± 0.6	1 ± 0.5	1 ± 0.6	0.844
NLR		5 ± 6	4 ± 3.4	10 ± 10.4	0.0001 **
Hb (d/dL)		13 ± 1.8	13 ± 1.7	13 ± 2.4	0.18
PLT (mm^3^)		237 ± 96.5	244 ± 98.7	207 ± 84	0.389
LDH (UI/mL)		333 ± 159.1	309 ± 111.6	428 ± 265.8	0.025 *
D-Dimer (mcg/mL)		1 ± 0.8	1 ± 0.6	2 ± 1.3	0.002 **
Fibrinogen (mg/dL)		554 ± 136.4	552 ± 127.2	571 ± 141.5	0.583
INR		1 ± 0.1	1 ± 0.1	1 ± 0.2	0.485
CRP (mg/dL)		5 ± 4.6	5 ± 4.8	9 ± 5.8	0.439
PaO2 (kPa)		71 ± 13.8	73 ± 13.7	69 ± 22	0.053
SpO_2_ (%)		94 ± 3.5	95 ± 2.7	92 ± 4.6	0.003 **
PF		272 ±111	298 ± 101	170 ± 94	0.0001 **
Time symptoms-to-hospital		9 ± 5	9 ± 5	10 ± 10	0.591
Clinical observation time (days)		15 ± 11	15 ± 12	12 ± 5	0.059
***Quantitative lung CT***					
Lung volume (mL)		5000 ± 1547	4740 ± 1514	3538 ± 1344	0.02 *
Lung weight (g)		983 ± 237	995 ± 248	935 ± 189	0.459
Non-aerated tissue (weight, g)		45 ± 30	39 ± 22	69 ± 46	0.003 **
Poorly aerated tissue (weight, g)		192 ± 118	172 ± 87	271 ± 184	0.011 *
Well-aerated tissue (weight, g)		741 ± 22	778 ± 209	593 ± 21	0.011 *
Overinflated tissue (weight, g)		5 ± 5	5.4 ± 5	2.6 ± 3	0.068
NAw (%)		5 ± 3	4 ± 2	7 ± 5	0.001 **
NAv (%)		1 ± 2	1 ± 1	3 ± 3	0.003 **
HDw (%)		24 ± 13.2	22 ± 9	36 ± 22	0.001 **
HDv (%)		10 ± 10	8 ± 6	18 ± 17	0.002 **

CVD: cardiovascular disease; CKF: chronic kidney failure; COPD: chronic obstructive pulmonary disease; GI: gastrointestinal; NLR: neutrophile-to-leucocyte ratio; Hb: hemoglobin; PLT: platelet; LDH: lactate dehydrogenase; INR: international normalized ratio; CRP: C-reactive protein; PaO_2_: oxygen partial pressure; SpO_2_: peripheral oxygen saturation; PF: PaO_2_/FiO_2_; NAw (%): non-aerated weight percentage; NAv (%): non-aerated volume percentage; HDw (%): high-density weight percentage; HDv (%): high-density volume percentage * significant difference: level of significance: *p*-value < 0.05; ** significant difference: level of significance: *p*-value < 0.01.

**Table 2:** Pearson correlation between quantitative lung CT parameters and significantly different laboratory and respiratory findings.

		NLR	LDH	D-Dimer	PaO_2_	PF	SpO_2_
Non-aerated tissue (g)	r (Pearson)	0.657 **	0.373 **	0.329 *	−0.087	−0.353 **	−0.211
	*p*-value	0.0001	0.005	0.015	0.529	0.008	0.121
Poorly aerated tissue (g)	r (Pearson)	0.539 **	0.484 **	0.310 *	−0.13	−0.397 **	−0.178
	*p*-value	0.0001	0.0001	0.022	0.343	0.003	0.193
Well-aerated tissue (g)	r (Pearson)	−0.307 *	−0.192	−0.336 *	−0.251	0.088	0.011
	*p*-value	0.023	0.160	0.013	0.064	0.521	0.936
Overinflated tissue (g)	r (Pearson)	0.033	−0.112	0.009	0.085	0.005	−0.115
	*p*-value	0.812	0.415	0.951	0.535	0.974	0.402
NAw (%)	r (Pearson)	0.657 **	0.325 *	0.385 **	0.033	−0.302 *	−0.148
	*p*-value	0.0001	0.015	0.004	0.812	0.025	0.281
NAv (%)	r (Pearson)	0.659 **	0.358 **	0.513 **	0.155	−0.286 *	−0.041
	*p*-value	0.0001	0.007	0.0001	0.258	0.034	0.767
HDw (%)	r (Pearson)	0.637 **	0.434 **	0.434 **	0.032	−0.365 **	−0.133
	*p*-value	0.0001	0.001	0.001	0.816	0.006	0.333
HDv (%)	r (Pearson)	0.638 **	0.472 **	0.503 **	0.114	−0.365 **	−0.064
	*p*-value	0.0001	0.0001	0.0001	0.405	0.006	0.64

NLR: neutrophile-to-leucocyte ratio; LDH: lactate dehydrogenase; PaO_2_: oxygen partial pressure; SpO_2_: peripheral oxygen saturation; PF: PaO_2_/FiO_2_; NAw (%): non-aerated weight percentage; NAv (%): non-aerated volume percentage; HDw (%): high-density weight percentage; HDv (%): high-density volume percentage; * significant difference: level of significance: *p*-value < 0.05; ** significant difference: level of significance: *p*-value < 0.01.

**Table 3:** Cox regression analysis.

Univariate Analysis			Multivariate Analysis					
	HR		HR		HR		HR	
	(95% CI)	*p*	(95% CI)	*p*	(95% CI)	*p*	(95% CI)	*p*
Lung volume (mL)	0.99 (0.99–1)	0.037 *						
Lung weight (g)	1 (0.99–1.0)	0.89						
Non-aerated tissue (weight, g)	1.03 (1.01–1.1)	0.001 **	1.02 (1–1.05)	0.046 *				
Poorly aerated tissue (weight, g)	1.0 (1.0–1.01)	0.003 **	1.0 (0.995–1.01)	0.786				
Well-aerated tissue (weight, g)	0.995 (0.99–0.99)	0.012 *						
Overinflated tissue (weight, g)	0.85 (0.68–1.05)	0.137						
NAw (%)	1.4 (1.2–1.7)	0.0001 **			1.41( 1.03–0.53)	0.031 *	1.41 (1.05–1.9)	0.024 *
NAv (%)	1.55 (1.2–2.0)	0.001 **			0.98 (0.59–1.64)	0.943		
HDw (%)	1.07 (1.03–1.1)	0.0001 **						
HDv (%)	1.06 (1.02–1.1)	0.005 **						
NLR	1.1 (1.03–1.2)	0.004 **					0.94 (0.81–1.09)	0.403
LDH	1 (1–1.004)	0.118						
D-Dimer	2.54 (1.32–4.9)	0.005 **					1.94 (0.78–4.79)	0.153
PF	0.991 (0.98–999)	0.037 *					0.996 (0.99–1.01)	0.48

NAw (%): non-aerated weight percentage; NAv (%): non-aerated volume percentage; HDw (%): high-density weight percentage; HDv (%): high-density volume percentage; NLR: neutrophile-to-leucocyte ratio; LDH: lactate dehydrogenase; PF: PaO_2_/FiO_2_; * significant difference: level of significance: *p*-value < 0.05; ** significant difference: level of significance: *p*-value < 0.01.

## Data Availability

Data Available on request.
